# Prevalence of Gestational Diabetes Mellitus among pregnant women attending antenatal care clinic of St. Paul’s Hospital Millennium Medical College, Addis Ababa, Ethiopia

**DOI:** 10.1186/s40842-022-00139-w

**Published:** 2022-02-23

**Authors:** Balkachew Nigatu, Tigist Workneh, Thomas Mekuria, Helen Yifter, Yeshiwondim Mamuye, Addisu Gize

**Affiliations:** 1 Department of Obstetrics and Gynecology, St. Paul Hospital Millennium Medical College, Addis Ababa, Ethiopia; 2 Department of Internal Medicine, St. Paul Hospital Millennium Medical College, Addis Ababa, Ethiopia; 3 Endocrinology Unit, Department of Internal Medicine, College of Health Science, Addis Ababa University, Addis Ababa, Ethiopia; 4 Department of Microbiology, St. Paul Hospital Millennium Medical College, Addis Ababa, Ethiopia

**Keywords:** Gestational Diabetes Mellitus, Prevalence, One-step 75-g Oral glucose tolerance test, Cross sectional study, Logistic regression, Ethiopia

## Abstract

**Background:**

The prevalence of Gestational Diabetes Mellitus (GDM) varies worldwide among racial and ethnic groups, population characteristics (eg, average age and body mass index (BMI) of pregnant women), testing method, and diagnostic criteria. This study was aimed at determining the prevalence of GDM using the one-step 75-g Oral glucose tolerance test (OGTT) protocol, with plasma glucose measurement taken when patient is fasting and at 1 and 2 h and identify associated risk factors among pregnant women attending antenatal care clinic at St. Paul Hospital Millennium Medical College (SPHMMC) in Addis Ababa, Ethiopia.

**Methods:**

Institution based cross sectional study was conducted from April, 2017 to October, 2017 at antenatal care clinic of SPHMMC among a randomly selected sample of 390 eligible pregnant women. Data were collected using a pretested questioner using 5% of the total sample size and later was modified accordingly to capture all the necessary data. Descriptive statistics, independent t-test and Binary Logistic Regression were used for analysis using SPSS version 23.0.

**Results:**

The prevalence of GDM among the study population was 16.9%. Factors that affect prevalence of GDM were age group (AOR = 2.75, 95% CI: 1.03, 7.35 for 30–34 years old and AOR = 4.98, 95% CI: 1.703, 14.578 for ≥ 35 years old)and BMI (AOR = 2.23, 95% CI: 1.21, 4.11).

**Conclusions:**

The prevalence of GDM among the study population is higher than previous reports in Ethiopia and even in other countries. This implies that these women and their newborns might be exposed to increased risk of immediate and long term complications from GDM including future risk of GDM and Type II Diabetes Mellitus.

**Supplementary Information:**

The online version contains supplementary material available at 10.1186/s40842-022-00139-w.

## Background

Diabetes is a complex metabolic disorder characterized by chronic hyperglycemia. There are different types of diabetes: Type I Diabetes Mellitus (TIDM), Type II Diabetes Mellitus (TIIDM) and Gestational Diabetes Mellitus (GDM). The number of people with diabetes is steadily increasing globally in recent decades. Global prevalence of diabetes has doubled since 1980 from 4.7% to 8.5% in 2016. The prevalence is growing most rapidly in low- and middle-income countries. Associated risk factors such as being overweight or obese are also increasing [1, 2]. GDM, one of the most common medical complications during pregnancy, is defined as carbohydrate intolerance of variable degree with an onset or first recognition occurring during pregnancy and some factors are believed to increase the risk of developing it [3, 4].

The consequences of unmanaged GDM in pregnancy can be severe both to the mother and the newborn and includes an increased risk for Preeclampsia, hydraminos, fetal macrosomia, fetal organomegaly, birth trauma, caesarean section, obstructed labor, perinatal mortality, neonatal respiratory problems and metabolic complications (hypoglycemia, hyperbilirubinemia, hypocalcemia), increased risks of miscarriage and congenital anomalies which can be especially serious in low-resource settings [3, 5].

The prevalence of gestational diabetes varies worldwide among racial and ethnic groups, population characteristics (eg, average age and body mass index of pregnant women), testing method, and diagnostic criteria. Prevalence has been increasing over time, possibly related to increases in mean maternal age, weight and other factors [1].

It is estimated by International Diabetes Federation (IDF) that 21.3 million or 16.2% of live births to women in 2017 had some form of hyperglycaemia in pregnancy. An estimated 86.4% of those cases were due to GDM, 6.2% due to diabetes detected prior to pregnancy, and 7.4% due to other types of diabetes (including TIDM and TIIDM) first detected in pregnancy [6].

The majority (88%) of cases of hyperglycaemia in pregnancy were reported in low- and middle-income countries, where access to maternal care is often limited. In Africa, number of live births affected by hyperglycaemia in pregnancy in women aged 20–49 years in 2017 was 3.4 million, age-adjusted prevalence of hyperglycaemia in pregnancy was 9.5% and raw prevalence was 10.4% [1, 6].

A systematic review of literatures in 2016 shows that direct comparisons of GDM burden across countries or regions are challenging given the great heterogeneity in screening approaches, diagnostic criteria, and underlying population characteristics [7].

A systematic review of literature in Africa showed there are few studies on prevalence and risk factors for GDM in Sub-Saharan Africa and heterogeneity is high. Prevalence was up to about 14% when high-risk women were studied [8]. Another systematic review and metanalysis of African GDM cases showed that the pooled prevalence of GDM was 13.61% and it was 14.28% in the sub-Saharan African region. And the study also showed that overweight and obesity, macrosomia, family history of diabetes, history of stillbirth, history of abortion, chronic hypertension and history of previous GDM were positively associated with GDM [9].The prevalence of GDM in South Africa, as assessed by a 2-h oral OGTT with blood collected at 0, 30 and 120 min, reported to range between 1.6–8.8% [10]. From studies conducted in Nigeria, one of the four studies compared the detection rate of the three-hour 75 g Oral glucose tolerance test (OGTT) using the World Health Organization (WHO) 1985 criteria to the three-hour 100 g OGTT using the National Diabetes Data Group (NDDG) criteria. The 75 g OGTT with WHO 1985 diagnostic criteria yielded a higher GDM prevalence (11.6% versus 4.5%) [11].

A community based study conducted at Tigray administrative region, northern Ethiopia,among a total of 890 pregnant women with gestational age of 24 weeks and above using 75 gm OGTT as the diagnostic test based on the WHO 1985 criteria shows that the prevalence rate of gestational diabetes mellitus was found to be 3.7% [12].

Though there are no recent studies on prevalence of GDM in Ethiopia, it is believed that the prevalence of GDM varies in direct proportion to the prevalence of type 2 DM in a given population. In 2017 close to 46% of all adults with diabetes in Africa lived in four major countries, Ethiopia being top on the list with 2.6 million adults with TIIDM. Therefore, it indirectly shows that the prevalence of GDM might have also increased leading to increased risk of maternal and newborn complications [3].

Therefore, the objective of this study was to determine the prevalence of GDM and identify associated risk factors among pregnant women attending antenatal care clinic of St. Paul’s Hospital Millennium Medical College (SPHMMC) in Addis Ababa, Ethiopia so that, risk factor targeted intervention can be given.

## Methods

### Study design and subjects

The study design was institution based cross sectional study and was conducted at antenatal care (ANC) clinic of SPHMMC from October to December 2017. At the time of the study, the hospital has annual delivery rate of 9600. Its outpatient department attends 60–70 antenatal cases daily.

### Source and study population

The source population was all pregnant women who were on follow-up at ANC clinic of SPHMMC from October to December 2017. The gestational age was calculated using Last normal menstrual period or early ultrasound.

The study population was all selected pregnant women who were on follow-up at ANC clinic of SPHMMC who fulfill the inclusion criteria.

Those who are > 18 years old and with gestational age between 24 and 28 weeks based on reliable date or early ultrasound estimation done by a trained professional – radiologist, radiographer, or obstetrician were included. Those women with unknown gestational age and those on steroids were excluded.

### Sample size determination and sampling procedure

The sample size was determined using single population proportion formula by considering the following statistical assumptions: 95% confidence interval (CI), 50% proportion (as there are no recent studies) and 5% marginal error and 10% non-response rate. The final sample size for this study was 422.

Among the pregnant women who visited the ANC clinic during the study period, simple random sampling method using table of random numbers was employed to select the study participants.

### Operational definition

#### Gestational diabetes

It was diagnosed using the One-step strategy by performing a 75-g Oral glucose tolerance test (OGTT) protocol, with plasma glucose measurement taken when patient is fasting and at 1 and 2 h, at 24–28 weeks of gestation in women not previously diagnosed with overt diabetes. The OGTT was performed in the morning after an overnight fast of at least 8 h [13, 14].

Subsequently, the diagnosis of GDM was made when any of the following plasma glucose values were met or exceeded:

- Fasting: 92 mg/dL (5.1 mmol/L)
- 1 h: 180 mg/dL (10.0 mmol/L)
- 2 h: 153 mg/dL (8.5 mmol/L)

### Data collection

Pre-tested questionnaire and data abstraction tool that consists of questions to assess the relevant variables was used to collect the necessary data from the pregnant women and their medical charts by trained data collectors. Laboratory test was conducted to assess the GDM status using the one-step 2-h 75 gm OGTT.

### Data management and statistical analysis

The collected data was cleaned, coded and entered into Epi-Info version 7.2.1.0, and exported to SPSS version 23.0 software for analysis. Participants’ socio-demographic characteristics, personal and co-morbid illness related factors and obstetric related factors are presented using the relevant descriptive statistics.

Univariate analysis was performed at 25% level of significance to screen out potentially significant independent variables. The association between the dependent variable and independent variables were analyzed using Binary Logistic Regression using the significant and relevant independent variables.The adequacy of the final model was checked using the Hosmer and Lemeshow goodness of fit test and the final model fitted for the data well (x^2^_(8)_ = 11.771 and *p*-value = 0.162).For Binary Logistic Regression, 95% confidence interval was calculated and variables with *p*-value ≤ 0.05 were considered as statistically significant.

## Results

### Socio-demographic variables

From the 422 samples, information was collected from 390 pregnant women making the response rate 92.4%. Majority of the pregnant women (40.3%) were in the age range of 25–29 years, 62.6% of them were from Addis Ababa and majority were married (97.4%). More than half (66.2%) of the pregnant women were Orthodox. More than a third (35.1%) were secondary school complete and close to 90% of the participants had monthly income of < 5000 Ethiopian Birr (ETB) (< 1500 ETB (44.1%) and 1500–5000 ETB (43.8%)) (Table 1).

**Table 1:** Socio–demographic variables among pregnant women on ANC follow up, Addis Ababa, 2018 (*n* = 390)

Variable	Frequency	Percent (%)
**Age group (in years)**		
18–24	98	25.1
25–29	157	40.3
30–34	87	22.3
> = 35	48	12.3
**Place of residence**		
Addis Ababa	244	62.6
Outside Addis Ababa	146	37.4
**Marital status**		
Married	380	97.4
Single or Divorced	10	2.6
**Religion**		
Orthodox	258	66.2
Muslim	83	21.3
Protestant	49	12.6
**Education**		
No formal education	52	13.3
Primary school complete	127	32.6
Secondary school complete	137	35.1
College graduate	74	19.0
**Monthly income (in ETB)**		
< 1500	172	44.1
1500–5000	171	43.8
> 5000	47	12.1

### Personal and co-morbid illness related variables

Regarding diabetes related history, 18 (4.6%) of the pregnant women had positive first degree family history of diabetes and 33 (8.5%) of them had history of hypertension. Majority of the pregnant women (69.2%) had a BMI of < 25 kg/m^2^. Regarding HIV status, majorities (93.1%) were non-reactive, 4.1% were reactive and the rest (2.6%) did not receive the test.

Regarding blood pressure measured during the interview time, the mean systolic and diastolic pressure among the pregnant women were 108.45 +—12.32 SD and 68.70 +—9.00 SD respectively. The 2 h 75 gm OGTT result showed that the mean +—SD for the FBS, 1 h and 2 h glucose values were 83.23 +—11.74, 116.97 + -42.79 and 100.38 +—27.69 respectively (Table 2).

**Table 2:** Personal and Co-morbid illness related variables among pregnant women on ANC follow up, Addis Ababa, 2018 (*n* = 390)

Variable	Frequency (%) or Mean ± Standard deviation
**Family history of DM**	
No	372 (95.4%)
Yes	18 (4.6%)
**History of hypertension**	
No	357 (91.5%)
Yes	33 (8.5%)
**BMI (in Kg/m**^**2**^**)**	
< 25	270 (69.2%)
> = 25	120 (30.8%)
**HIV status**	
Reactive	16 (4.1%)
Non-reactive	364 (93.3%)
Not tested	10 (2.6%)
**Systolic blood pressure (mmHg)**	108.45 ± **12.32**
**Diastolic blood pressure (mmHg)**	68.70 ± **9.0**
**Fasting blood sugar (mg/dl)**	83.23 ± **11.74**
**1 h post prandial glucose (mg/dl)**	116.97 ± **42.79**
**2 h post prandial glucose (mg/dl)**	100.38 ± **27.69**

### Obstetric related variables

Regarding obstetric history, majority were multigravida (71.0%) and multiparous (66.4%). Less than one-third (28.2%) had a history of bad obstetric outcome and only 4 (1.0%) had a history of GDM in previous pregnancy. Among the 4 pregnant women with previous history of GDM, 2 has developed GDM in the current pregnancy (Table 3).

**Table 3:** Obstetric related variables among pregnant women on ANC follow up, Addis Ababa, 2018 (*n* = 390)

Variable	Frequency	Percent (%)
**Gravidity**		
Primigravida	113	29.0
Multigravida	227	71.0
**Parity**		
Nulliparous	131	33.6
Multiparous	259	66.4
**History of bad obstetric outcome**		
No	280	71.8
Yes^a^	110	28.2
**Previous history of GDM**		
No	386	99.0
Yes	4	1.0

Yes^a^: includes abortion, preterm delivery, still birth and early neonatal death

### Prevalence of GDM

Among the 390 pregnant women participated in the study, 66 (16.9%: 95% CI: 13.3, 20.8) had GDM and 324 (83.1%: 95% CI: 79.2, 86.7) did not have GDM.

### Factors associated with prevalence of GDM

From univariate analysis of the independent variables, age group, place of residence, educational level, monthly income, gravidity, parity, history of hypertension and BMI were significantly associated with development of GDM among pregnant women at 25% level of significance.

However, only age group and BMI were found to be significantly associated with development of GDM among pregnant women in Multiple Logistic Regression model at 5% level of significance.

Accordingly, after adjusting for other covariates, compared to those in the age range of 18–24 years, the odds of developing GDM among pregnant women in the age group 30–34 and >  = 35 years were 2.753 times and 4.982 times, respectively (AOR = 2.75, 95% CI: 1.03, 7.35 for 30–34 years old and AOR = 4.98, 95% CI: 1.70, 14.58 for ≥ 35 years old). On the other hand, pregnant women in the age group of 25–29 years did not show significant difference compared to those 18–24 years of age.

The odds of developing GDM among pregnant women with BMI of ≥ 25 kg/m^2^ were 2.23 times the odds of those with BMI of < 25 kg/m^2^ (AOR = 2.23, 95% CI: 1.21, 4.11). In other words, pregnant women with BMI of ≥ 25 kg/ m^2^ were 123.3% more likely to develop GDM compared with those with BMI of < 25 kg/ m^2^ (Table S1).

## Discussion

The main purpose of this study was to determine the prevalence of GDM and identify associated factors among pregnant women attending ANC clinic of SPHMMC. The study revealed that 66 (16.9%) of the mothers developed GDM. Compared to a community based study conducted in other part of Ethiopia in 1999, the prevalence of GDM was significantly greater (3.7% in Tigray region Vs 16.9%). This difference can be accounted for the difference in the study area and study period, the current study is conducted in a tertiary hospital where there is a large flow of cases especially those with co-morbidity and complications like GDM. This increase could also be because of the increase in the risk factors responsible for GDM on a global scale including obesity and old maternal age especially in urban area. Also the two studies used different diagnostic criteria for diagnosing GDM, the criteria we used is a sensitive one that picks most pregnant with GDM. There is also a marked difference to a study conducted in Rwanda and South Africa which shows a prevalence of 8.3 and 1.6–8.8%, respectively. This difference could be could be because of the difference in the diagnostic criteria used to diagnose GDM in addition to the difference in study areas and population characteristics [10, 12]. The prevalence was, however, comparable with studies conducted in Nigeria and Saudi Arabia and a systematic review and meta-analysis in Africa [9, 11, 15].

The identified prognostic factors of this study are found to be analogous with literatures on the topic.

The age of patients is found to be an important factor that determines the prevalence of GDM. The study shows that the prevalence of GDM doesn’t show significant difference between 18–24 and 25–29 years of age. On the other hand, the odds of developing GDM is higher among pregnant women ≥ 35 years followed by the age group 30–34 years compared to women in 18–24 years age group indicating that for pregnant women 30 years and older, the odds of developing GDM increases with age. This could be because, it is believed that as age increases the risk of developing chronic illnesses including diabetes increases. And as an individual develops one chronic illness the risk of developing another chronic illness increases which increases the risk to a higher level [1, 3, 6, 14, 16, 17].

In addition, the study found that BMI is another important factor that affects prevalence of GDM. The odds of developing GDM among pregnant women with BMI of ≥ 25 kg/m^2^ were 2.233 times the odds of those with BMI of < 25 kg/m^2^. This is because, obesity is very known risk factor for other chronic illnesses including diabetes mellitus especially as part of the metabolic syndrome. Therefore, as weight of the pregnant women increases the risk of insulin resistance increases which in turn increases the risk of developing GDM [1, 3, 6, 9, 14, 16, 17].

## Conclusion

The prevalence of GDM among pregnant women attending ANC clinic of SPHMMC was 16.9%. This finding is higher than previous reports in Ethiopia and even in other countries. This implies that these women and their newborns are exposed to increased risk of immediate and long term complications from GDM including future risk of GDM and T2DM to the mother.

Our study showed that major factors that affect prevalence of GDM were age group and BMI. Pregnant women 30 years and older and those women who were above the normal weight (overweight, obese and morbidly obese) were found to have higher risk of developing GDM.

On the other hand, the results of this study indicated that the prevalence of GDM is not statistically different among groups classified by place of residence, marital status, religion, education, monthly income, family history of DM, history of hypertension, HIV status, gravidity, parity, history of bad obstetric outcome and previous history of GDM.

Therefore, from the findings of this study, we recommend the following:

- To give special attention for those pregnant women whose age is 30 years and above and whose BMI is above the normal range during ANC follow-up.
- Screening of GDM should be part of routine ANC follow up for mothers. Health facilities can at least do FBS at the first ANC visit and 24–28 weeks of pregnancy.
- Professional societies and the Ministry of Health need to develop a protocol on screening of mothers for GDM.
- To conduct further study including additional relevant personal (behavioral) factors.

## Supplementary Information

**Additional file 1:** **Table S1.** GDMManuscript.

## Data Availability

The datasets used and/or analyzed during the current study are available from the corresponding author up on reasonable request.

## Abbreviations

ANC: Antenatal Care
AOR: Adjusted Odds Ratio
BMI: Body Mass Index
CI: Confidence Interval
CIRHT: Center for International Reproductive Health Training
FBS: Fasting Blood Sugar
GDM: Gestational Diabetes Mellitus
IDF: International Diabetes Federation
IRB: Institutional Review Board
NDDG: National Diabetes Data Group
OGTT: Oral Glucose Tolerance Test
OR: Odds Ratio
SPSS: Statistical Package for Social Science
T2DM: Type 2 Diabetes Mellitus
WHO: World Health Organization
