# Supplementary material for: Prevalence of Gestational Diabetes Mellitus among pregnant women attending antenatal care clinic of St. Paul’s Hospital Millennium Medical College, Addis Ababa, Ethiopia
Source: Clin Diabetes Endocrinol. 2022 Feb 23;8:2. doi: 10.1186/s40842-022-00139-w (PMC8867668; doi:10.1186/s40842-022-00139-w)
Supplement: Supplementary file 1 — Additional file 1: Table S1. GDMManuscript. [file 40842_2022_139_MOESM1_ESM.docx]

**Additional file _Table 4_GDM Manuscript**

**Table 4:** Univariate and Multivariable analysis for factors associated with development of GDM among pregnant women on ANC follow up, Addis Ababa, 2018 (n=390)

| **Variables** | **GDM** | | **COR(95% CI)** | **AOR(95% CI)** | **p-value** |
| --- | --- | --- | --- | --- | --- |
|  | **No** | **Yes** |  |  |  |
| **Age group (in years)** |  |  |  |  |  |
| 18-24 | 90 | 8 | 1.00 | 1.00 | **0.021*** |
| 25-29 | 134 | 23 | 1.931 (0.827,4.507) | 1.799 (0.730,4.436) |  |
| 30-34 | 67 | 20 | 3.358 (1.395,8.087) | **2.753 (1.031,7.347)** |  |
| >=35 | 33 | 15 | 5.114(1.985,13.173) | **4.982 (1.703,14.578)** |  |
| **Place of residence** |  |  |  |  |  |
| Addis Ababa | 198 | 46 | 1.00 | 1.00 | 0.547 |
| Outside Addis Ababa | 126 | 20 | 0.683 (0.386,1.209) | 0.827 (0.447,1.532) |  |
| **Education** |  |  |  |  |  |
| No formal education | 43 | 9 | 1.00 | 1.00 | 0.105 |
| Primary school | 114 | 13 | 0.545 (0.217,1.366) | 0.805 (0.300,2.157) |  |
| Secondary school | 111 | 26 | 1.119 (0.485,2.581) | 1.729 (0.675,4.428) |  |
| College graduate | 56 | 18 | 1.536 (0.629,3.752) | 2.307 (0.786,6.770) |  |
| **Monthly income** |  |  |  |  |  |
| <1500 | 142 | 30 | 1.00 | 1.00 | 0.269 |
| 1500-5000 | 147 | 24 | 0.773 (0.431,1.386) | 0.619 (0.316,1.213) |  |
| >5000 | 35 | 12 | 1.623 (0.755,3.487) | 1.053 (0.413,2.683) |  |
| **Gravidity** |  |  |  |  |  |
| Primigravida | 97 | 16 | 1.00 | 1.00 | 0.336 |
| Multigravida | 227 | 50 | 1.335 (0.725,2.460) | 0.565 (0.177,1.808) |  |
| **Parity** |  |  |  |  |  |
| Nulliparous | 113 | 18 | 1.00 | 1.00 | 0.740 |
| Multiparous | 211 | 48 | 1.428 (0.793,2.571) | 1.207 (0.397,3.665) |  |
| **Hypertension** |  |  |  |  |  |
| No | 302 | 55 | 1.00 | 1.00 | 0.127 |
| Yes | 22 | 11 | 0.364 (0.167,0.794) | 0.512(0.217,1.211) |  |
| **BMI** |  |  |  |  |  |
| < 25 | 238 | 32 | 1.00 | 1.00 | **0.010*** |
| >= 25 | 86 | 34 | 2.940 (1.710,5.056) | **2.233 (1.214,4.109)** |  |

**Note:** COR, Crude odds ratio; AOR, Adjusted odds ratio; CI, Confidence interval; ***** Statistically significant

Table 4: Univariate and Multivariable analysis for factors associated with development of GDM among pregnant women on ANC follow up, Addis Ababa, 2018 (n=390)
